# High-throughput optimization of antibody production in CHO cells by tuning heavy- and light-chain promoter strength

**DOI:** 10.3389/fbioe.2025.1747473

**Published:** 2026-01-09

**Authors:** Marzia Rahimi, Anna Christina Adams, Lise M. Grav, Lars K. Nielsen, Jesús Lavado-García

**Affiliations:** 1 The Novo Nordisk Foundation Center for Biosustainability, Technical University of Denmark, Kongens Lyngby, Denmark; 2 Autralian Institute for Bioengineering and Nanotechnology, The University of Queensland, Brisbane, QLD, Australia

**Keywords:** antibody production, CHO cell, heavy chain (HC), highthroughput screening, light chain (LC), promoter strength

## Abstract

Monoclonal antibody (mAb) production in CHO cells depends, among other factors, on balanced co-expression of the heavy (HC) and light (LC) chains. Imbalances between HC and LC can reduce titer, compromise product quality, and negatively affect cell viability, which means that the optimal LC/HC expression ratio should be identified as early as possible in cell line development. However, systematically testing multiple LC/HC expression ratios for many antibody candidates using traditional workflows is slow and resource intensive. Here, we present a high-throughput screening platform, coupled with a design-of-experiments (DoE) strategy, to identify optimal LC/HC expression balance at the transient stage. The system uses single-vector constructs encoding both LC and HC under promoters of defined low, medium, or high strength, enabling combinatorial testing of LC/HC promoter pairs. We applied this workflow to three different antibodies and quantified titer, viable cell density, and viability 72 h post-transfection. The optimal LC/HC promoter ratio was antibody specific. For two antibodies, high LC combined with medium HC expression yielded the highest titer while maintaining cell viability. High LC with high HC expression (LC100–HC100) also produced high titer but caused reduced viable cell density and viability. For the third antibody, the best-performing configuration was medium LC with medium HC, with medium LC and high HC as a close second. Across all three antibodies, low-strength promoters for either chain consistently resulted in poor titer. Overall, this platform offers a rapid and scalable approach to define antibody-specific LC/HC promoter strength combinations that maximize productivity without compromising cell health, enabling more informed construct selection before committing to stable clone generation.

## Introduction

The global demand for monoclonal antibodies (mAbs) continues to grow rapidly, accounting for more than 80% of all biologics produced in mammalian cells (Walsh and Walsh, 2022; Crescioli et al., 2024). For their biopharmaceutical production, Chinese hamster ovary (CHO) cells remain the preferred expression system for large-scale biomanufacturing due to their capacity for high protein yields, proper protein folding, and human-compatible post-translational modifications. Developing efficient and robust CHO cell lines is therefore a key step in ensuring consistent antibody productivity and product quality across biomanufacturing pipelines. A balanced co-expression of the antibody heavy (HC) and light chains (LC) is essential for the proper assembly and secretion of functional antibodies. For instance, since the HC CH1 domain requires LC for proper folding, higher LC expression has been reported to enhance antibody assembly and secretion (Feige et al., 2009). However, an imbalance between HC and LC expression can lead to the accumulation of unassembled or misfolded intermediates, formation of aggregates, and secretion of incomplete antibody species (Stoyle et al., 2017; Li et al., 2022; Yang et al., 2022). Such outcomes can reduce overall yields, affect glycosylation patterns, and complicate downstream purification. Consequently, the ratio between HC and LC transcription and translation is a critical determinant of both the quantity and quality of the final antibody product (Li et al., 2022).

Moreover, the ideal HC/LC expression balance can vary significantly depending on the antibody (Schlatter et al., 2005), making it impractical to rely on a single standardized configuration. Therefore, a rapid, systematic and high-throughput screening method capable of evaluating multiple promoter strength combinations at the transient stage is essential to identify the most productive ratio before proceeding to stable cell line generation. Such approaches not only accelerate the identification of optimal expression constructs but also enhance the reproducibility of cell line development workflows.

In this study, we present a high-throughput screening platform to evaluate different combinations of heavy and light chain promoter strengths for three antibodies in CHO cells. Our method integrates high-throughput plasmid cloning, transient transfection, expression quantification, and design of experiments (DoE) to systematically identify promoter ratios that maximize antibody yield. This approach allows efficient determination of the optimal expression cassettes configuration prior to stable cell line generation. Beyond antibody manufacturing, the workflow is adaptable to other recombinant protein expression systems where balancing multi-chain or multi-subunit protein expression is critical. By implementing this high-throughput strategy, researchers and process developers can significantly reduce development timelines, improve product consistency, and enhance the overall efficiency of mammalian-based bioproduction processes.

## Materials and equipment

### Molecular cloning

- PCR primers for amplification of backbones and stuffer plasmids
- PCR primers for amplification of antibody variable regions
- Phusion High-Fidelity DNA Polymerase Master Mix (M0530S, New England Biolabs, Ipswich, MA, United States)
- Nuclease-free water (AM9906, Thermo Fisher Scientific, Waltham, MA, United States)
- NucleoSpin Gel and PCR Clean-up Kit (740609.250, Macherey-Nagel, Düren, Germany)
- GeneRuler 1 kb DNA Ladder (SM0311, Thermo Fisher Scientific, Waltham, MA, United States)
- 1% agarose gel (1 g agarose powder dissolved in 100 mL of 1× TAE buffer)
- SYBR Safe DNA Gel Stain (S33102, Thermo Fisher Scientific, Waltham, MA, United States)
- FastDigest restriction enzymes: *EcoRI* (FD0274), *BamHI* (FD0054), *SalI* (FD0644), and *KpnI* (FD0524) (Thermo Fisher Scientific, Waltham, MA, United States)
- NEBuilder HiFi DNA Assembly Master Mix (E2621L, New England Biolabs, Ipswich, MA, United States)
- QIAGEN Plasmid Plus 96 Miniprep Kit (16181, QIAGEN, Venlo, The Netherlands)

### Bacterial culture

- *Escherichia coli* Mach1 T1R chemically competent cells (C869601, Thermo Fisher Scientific, Waltham, MA, United States)
- 48 well LB agar plates (prepared in-house and supplemented with 60 μg/mL ampicillin)
- 2× YT medium: 16 g tryptone, 10 g yeast extract, and 5 g NaCl per liter of distilled water; pH adjusted to 7.0 and sterilized by autoclaving (121 °C, 20 min)
- SOC medium: prepared according to standard formulations (2% tryptone, 0.5% yeast extract, 10 mM NaCl, 2.5 mM KCl, 10 mM MgCl_2_, 10 mM MgSO_4_, and 20 mM glucose)
- Ampicillin (60 μg/mL, final concentration in liquid culture)

### Mammalian cell culture and transfection

- CHO-S cells (R80007, Life Technologies, Waltham, MA, United States)
- CD CHO medium (10743029, Gibco, Thermo Fisher Scientific, Waltham, MA, United States)
- L-Glutamine (25030081, Thermo Fisher Scientific, Waltham, MA, United States)
- Freestyle MAX Transfection Reagent (16447100, Thermo Fisher Scientific, Waltham, MA, United States)
- OptiPRO serum free medium (12309019, Thermo Fisher Scientific, Waltham, MA, United States)
- Transfection positive control expressing GFP (pMax-E2F1, 16007, Addgene, Watertown, MA, United States)
- Solution 18 (910-3018, ChemoMetec A/S, Allerød, Denmark)
- NC-Slide A8 (941-0002, ChemoMetec A/S, Allerød, Denmark)

### Plasticware and equipment

- MicroAmp Fast 96-Well Reaction Plate (4346907, Applied Biosystems, Thermo Fisher Scientific, Waltham, MA, United States)
- SimpliAmp Thermal Cycler (A24811, Applied Biosystems, Thermo Fisher Scientific, Waltham, MA, United States)
- Filtered pipette tips (10 μL, 200 μL, and 1000 μL; Thermo Fisher Scientific, Waltham, MA, United States)
- 1.5 mL and 2.0 mL microcentrifuge tubes (Eppendorf SE, Hamburg, Germany)
- Benchtop microcentrifuge (Eppendorf SE, Hamburg, Germany)
- Eppendorf Centrifuge 5810 R (Eppendorf SE, Hamburg, Germany)
- Gel electrophoresis system (Thermo Fisher Scientific, Waltham, MA, United States)
- 125 mL Erlenmeyer flasks with vent caps (431143, Corning Inc., Corning, NY, United States)
- 96-half-deep-well microplate (CR1496c, Enzyscreen B.V., Haarlem, The Netherlands)
- Autoclaved low-evaporation sandwich Duetz covers (CR1296a, Enzyscreen B.V., Heemstede, The Netherlands)
- Octet RED96 System (Sartorius, Göttingen, Germany)
- Protein A Biosensors (18-5010, Sartorius, Göttingen, Germany)
- Black 96-Well Microplates, Non-Binding Surface (3694, Corning Inc., Corning, NY, United States)
- Shaking incubator (37 °C, 350 rpm)
- Eppendorf New Brunswick S41i CO_2_ incubator for mammalian cell culture (37 °C, 5% CO_2_, 70% humidity, 325 rpm, 2.5 cm orbital diameter)
- Amersham Imager 600 (AI600, Cytiva, Marlborough, MA, United States)
- NanoDrop 2000 Spectrophotometer (Thermo Fisher Scientific, Waltham, MA, United States)
- NucleoCounter NC-200 (ChemoMetec A/S, Allerød, Denmark)
- CytoFLEX flow cytometer (Beckman Coulter, United States)
- Sterile disposable inoculating loop (10 μL; Deltalab, Barcelona, Spain)

### Software, online tools, and analytical services

- Sanger sequencing (Eurofins Genomics Ebersberg, Germany)
- GenSmart Codon Optimization Tool (GenScript, Piscataway, NJ, United States)
- GraphPad Prism (version 10.0, GraphPad Software, San Diego, CA, United States)
- CytExpert software (version 2.3, Beckman Coulter, United States)
- JMP Pro (Version18.2.1, SAS Institute Inc., Cary, NC, United States)

## Methods

### Antibodies

Antibodies B, C, and E used in this study correspond to previously described clones identified by phage display. Specifically, antibody B was originally denoted as 367-01-H01 (Laustsen et al., 2018), antibody C as 2551-01-A12 (Ledsgaard et al., 2023), and antibody E as TPL0004-01-A111, an unpublished clone for which the discovery and characterization methodology has been described by (Ahmadi et al., 2020). All three single-chain variable fragment (scFv) sequences were kindly provided by the Antibody Technologies Group, Department of Biotechnology and Biomedicine, Technical University of Denmark (DTU), Section for Biologics Engineering. The scFv sequences were subsequently reformatted into full-length immunoglobulin G (IgG) molecules using the Rituximab framework as the constant region backbone. Variable region sequences were codon-optimized for efficient expression in CHO cells using the GenScript optimization tool. The sequences of all antibodies variable region and the constant light (CL) and constant heavy (CH) chains are available in (Supplementary Table S1).

### Plasmid generation

The generation of plasmids encoding antibodies B, C, and E was carried out using a two-step cloning strategy. In the first step, backbone and stuffer plasmids were constructed, each containing the necessary elements required for antibody expression. In the second step, all DNA fragments corresponding to the antibody domains, the constant heavy (CH), constant light (CL), variable heavy (VH), and variable light (VL) regions, were assembled to generate the final expression plasmids.

The backbone plasmids contained all essential genetic elements required for replication in the bacterial host, insulator sequences, promoter, a signal peptide for the light chain, the constant region of the heavy chain, and a polyadenylation signal for the heavy-chain transcript (Supplementary Figure S1A–C).

The stuffer plasmids were designed to include, in addition to elements required for replication in the bacterial host, insulator sequences, promoter, a signal peptide for the heavy chain, the constant region of the light chain, and a polyadenylation signal for the light-chain transcript (Supplementary Figure S1D–F). DNA fragments for both the backbone and stuffer plasmids were generated by PCR amplification using in-house plasmids as templates. Three promoters of differing strength 5, 40, and 100 relative promoter units (RPUs), were used to evaluate expression performance. The promoter sequences, obtained from (Brown et al., 2017) are provided in (Supplementary Table S2).

The backbone plasmid was digested using *EcoRI* and *BamHI* restriction enzymes, the stuffer plasmid was digested using *KpnI* and *SalI* restriction enzymes, (Supplementary Figure S2) and purified by agarose gel electrophoresis followed by cleanup using the NucleoSpin Gel and PCR Clean-up Kit. The variable regions of antibodies were amplified by PCR and purified using the same cleanup procedure (Supplementary Figure S3). The concentrations of all purified DNA fragments were measured using Nanodrop 2000 spectrophotometer, and fragment dilutions were performed to ensure a 1:1 M ratio for assembly.

Assembly of all fragments, including backbone, stuffer, and variable regions was performed using the NEBuilder HiFi DNA Assembly Master Mix in MicroAmp Fast 96-Well Reaction Plates according to the manufacturer’s instructions. To enable high-throughput processing, the reaction volume was reduced to half of the standard protocol. Whereas the manufacturer’s protocol specifies a 20 µL reaction containing 10 µL of NEBuilder Master Mix and 10 µL of DNA fragments, our optimized workflow used a total volume of 10 μL, composed of 5 µL of NEBuilder Master Mix and 5 µL of total DNA.

Before transformation, *Escherichia coli* Mach1 competent cells were thawed on ice and distributed into 96-well reaction plates to a final volume of 10 µL per well. Following this, 1 µL of a 1:4 diluted assembled construct was used to transform the bacteria. The transformed bacteria were cultured in SOC media at 37 °C and 300 rpm in the same plate for 1 h. Subsequently, 20 µL from each well was transferred into a single well of a 48-well agar plate, and the culture was evenly spread using a sterile disposable inoculating loop. After overnight incubation at 37 °C, two colonies per condition were picked, and colony PCR was performed to identify correct assemblies. One verified clone per condition was then inoculated into a 96 deep-well plate and cultured overnight at 37 °C, 300 rpm. Endotoxin-free plasmid purification was performed using the QIAGEN Plasmid Plus 96 Miniprep Kit in accordance with the manufacturer’s guidelines. Plasmid concentration was measured using the Nanodrop 2000. Plasmid integrity was confirmed by Sanger sequencing.

### PCR amplification for NEBuilder cloning

All PCR reactions for NEBuilder cloning were performed using Phusion High-Fidelity DNA Polymerase master mix in 96 well plates. Thermal cycling conditions were as follows: initial denaturation at 98 °C for 30 s, followed by 35 cycles of denaturation at 98 °C for 10 s, annealing at 60 °C–65 °C for 30 s (adjusted according to primer Tm), and extension for 00:10–3:00 min (30 s/kb, adjusted according to fragment size), with a final extension at 72 °C for 10 min. PCR products were visualized by electrophoresis on a 1% agarose gel. All primers used are listed in (Supplementary Table S3).

### Cell culture

CHO-S suspension cells were cultured in CD CHO medium supplemented with 8 mM L-glutamine and maintained in 125 mL Erlenmeyer flasks with vent caps incubated at 37 °C, 5% CO_2_, 70% humidity and agitated at 130 rpm. Passaging occurred every 2–3 days, with cell growth and viability monitored using the NucleoCounter NC-200.

### High-throughput transient transfection

Prior to transfection, CHO-S cells were centrifuged at 200 × g for 5 min and resuspended in fresh CD CHO medium supplemented with 8 mM L-glutamine at a target viable cell density (VCD) of approximately 1 × 10^6^ cells/mL. The cell suspension was mixed thoroughly, and 230 µL was dispensed into each well of a 96-half-deep-well microplate. VCD and cell viability were measured using NucleoCounter NC-200 and plates were sealed with autoclaved, low-evaporation sandwich Duetz covers to minimize evaporation during incubation.

Antibody-encoding plasmids were diluted to a working concentration of 100 ng/μL in a 96-well plate to facilitate multichannel pipetting. For each transfection, a total of 312.5 ng DNA was transferred to a new 96-well plate and mixed with OptiPRO serum-free medium (SFM). In a separate plate, the appropriate amount of Freestyle MAX transfection reagent was diluted in OptiPRO SFM and incubated for 5 min. The transfection reagent mixture was then combined with the DNA mixture and incubated for an additional 6 min to allow complex formation. The resulting DNA–lipid complexes were added to the cell suspension, and the plates were incubated at 37 °C, 5% CO_2_, 70% humidity, and agitated at 325 rpm for 72 h. A transfection control using a plasmid encoding GFP was included as a positive control.

### Transfection efficiency

Transfection efficiency was determined 72 h post-transfection (72hpt) by quantifying GFP-positive cells using a CytoFLEX flow cytometer. Forward scatter (FSC) and side scatter (SSC) parameters were used to exclude debris and cell aggregates. The GFP signal was detected using the FITC channel (excitation 488 nm, emission 525/40 nm) (Supplementary Figure S4). Data were analyzed with CytExpert software. A gating strategy was established using non-transfected cells to define background fluorescence. The percentage of GFP-positive cells within the live-cell population was used as a measure of transfection efficiency.

### Evaluation of antibody production

Three days after transfection, the VCD was measured, and 200 µL of each culture was transferred to a new 96-well plate and centrifuged at 200 × g for 5 min. The supernatant was collected and transferred to a separate 96-well plate for antibody quantification.

Antibody concentrations in the culture supernatants were determined using the Octet RED96 system according to the manufacturer’s instructions. Protein A biosensors were pre-equilibrated in sample diluent buffer consisting of phosphate-buffered saline (PBS) containing 0.1% (w/v) bovine serum albumin (BSA) and 0.02% (v/v) Tween-20. Between measurements, biosensor tips were regenerated using 10 mM glycine (pH 1.7). The absolute antibody concentration in each sample was calculated from a standard curve generated using a serial dilution of Rituximab IgG.

### Design of experiment (DoE)

To identify the optimal balance between LC and HC expression, we used a Custom (D-optimal) design in JMP. Two categorical factors were studied, LC promoter strength and HC promoter strength, each at three levels corresponding to relative promoter units (RPUs): 5 (low), 40 (medium), 100 (high). The candidate set comprised all nine feasible LC × HC combinations. The *a priori* model specified main effects and their interaction (Equation 1).

Model:LC+HC+LC · HC (1)

Design generation followed the general linear model (Equation 2).

y=X β+ε, (2)

where X is the design matrix, β are model parameters, y the response and ε the error of the model.

The D-optimal algorithm selected the subset of candidate runs that maximizes the determinant of the information matrix (Equation 3), which is equivalent to minimizing the generalized variance of the least-squares estimators, because

$$Var\;\left(\;\beta \right)=\sigma^{2}{\left(X^{´}X\right)}^{-1}=>\;\det\left\{Var\left(\beta \right)\right\}\propto \det{\left(X^{´}X\right)}^{-1}$$ (3)

This yields the most precise coefficient estimates for the specified model, given the run budget and any constraints. The final plan contained N = 18 randomized experiments (Supplementary Tables S4–S6) (the 9 LC × HC combinations, each duplicated), providing 9 residual degrees of freedom for inference (Mitchell, 1974; Goos and Bradley, 2011; Rahman et al., 2022; Limmun et al., 2025). Responses were titer (mg/L), viable cell density (VCD; cells/mL), and viability (%). Each response was analyzed by ordinary least squares with LC and HC treated as nominal factors. Significance was evaluated using Type III ANOVA (Effect Tests) for LC and HC. Model fit was summarized by *R* ^2^ and Root Mean Square Error (RMSE). Model fit statistics and ANOVA effect tests are provided in (Supplementary Tables S7,S8).

## Results

### Plasmid generation and transfection in high-throughput system

**FIGURE 1:**
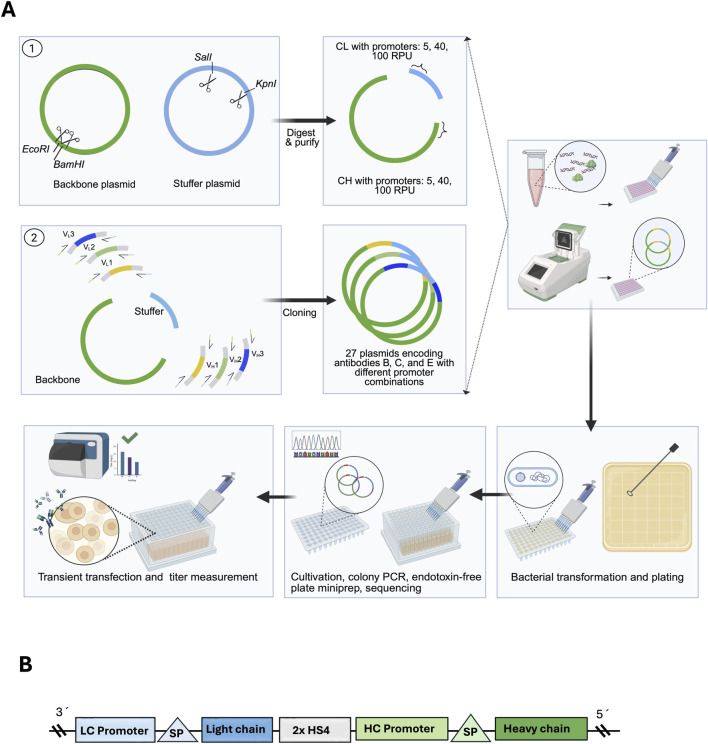
**(A)** High-throughput cloning and screening workflow. Backbone and stuffer plasmids were generated and digested, and the resulting DNA fragments together with PCR-amplified variable regions were assembled into a single vector encoding antibodies B, C, and E with different LC/HC promoter combinations. After bacterial transformation and plating, colonies were cultivated in 96-well plates, screened by colony PCR to confirm plasmid integrity, purified by endotoxin-free plate miniprep, and sequenced. Verified plasmids were then transiently transfected for antibody titer measurement. **(B)** Final plasmid design. Both light chain (LC) and heavy chain (HC) are expressed from the same construct, separated by two HS4 insulators.

To enable rapid screening of LC/HC promoter-strength ratios in CHO cells, we implemented a single-vector, high-throughput workflow in 96-well format. All steps, from PCR and cloning/colony PCR through miniprep, transfection, and antibody measurements, were performed in 96-well plates (Figure 1A). Each expression plasmid carried both antibody chains on one construct, including (i) the selected promoters (5, 40, or 100 RPU), (ii) signal peptides, (iii) the variable regions (mAbs B, C, and E), (iv) the constant light (CL) and constant heavy (CH) regions, and (v) a poly(A) signal and bacterial propagation elements. To minimize positional effects and transcriptional interference between cassettes, two insulating sequences were inserted between the LC and HC transcription units (Figure 1B). The synthetic promoters employed here have been previously validated in CHO cells, where their relative strength (5 < 40 < 100 RPU) was shown to correlate with transgene expression, including antibody heavy and light chain production, providing a rationale for their use to modulate LC/HC expression ratios in this workflow (Sou et al., 2023).

Plasmids were assembled in two steps. First, we generated stuffer and backbone plasmids carrying the CL and CH regions, respectively, and PCR-amplified the mAb-specific variable regions (VL and VH). Second, we performed a four-fragment assembly (CL, CH, VL, VH) to produce the final LC–HC dual-cassette constructs expressing antibodies B, C, and E. Assembly success was screened by colony PCR using a forward primer in the stuffer/CL region and a reverse primer in the backbone/CH region (Supplementary Figure S5), and confirmed by Sanger sequencing across junctions and variable regions. For each LC/HC promoter condition and antibody, one sequence-verified clone was selected for downstream experiments. In total, 27 plasmids were generated (3 antibodies × 9 LC/HC promoter combinations).

**FIGURE 2:**
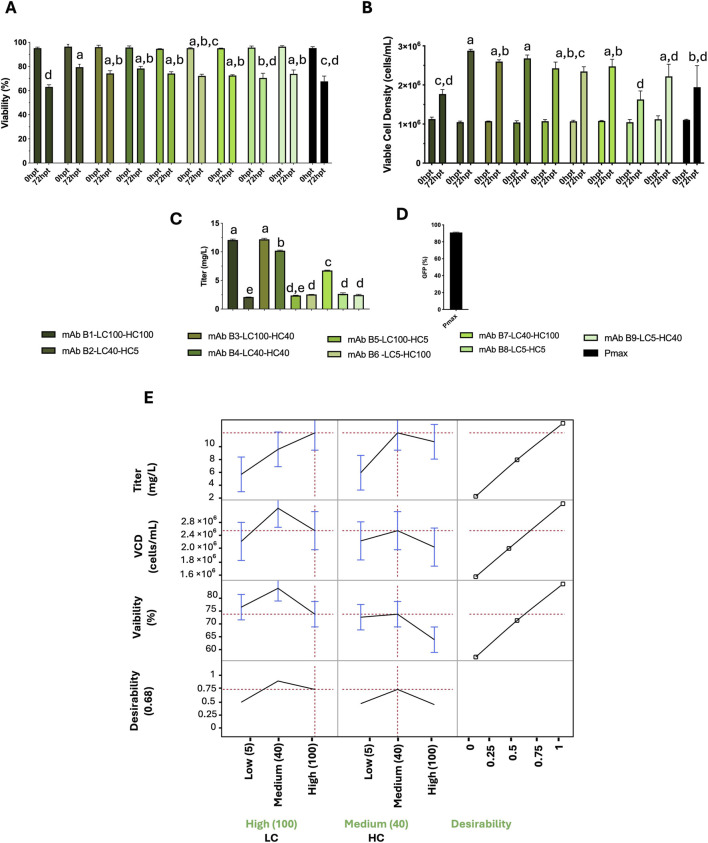
mAb B: effects of LC/HC promoter strength on titer, cell density, and viability post-transfection (72 hpt), and model profiling. **(A)** Viability (%), **(B)** viable cell density (VCD; cells/mL), and **(C)** titer (mg/L) for all LC/HC combinations. Promoter levels: 5, 40, and 100 RPU for both LC and HC. Bars show mean ± SD (n = 2). Statistical analysis was based on one-way ANOVA with Tukey's HSD; different letters indicate p < 0.05. **(D)** Transfection efficiency (% GFP+) of a control plasmid (pMAX-GFP) quantified by flow cytometry. **(E)** JMP profiler for least-squares models of titer, VCD, and viability versus LC/HC promoter strength. Left: predicted means with 95% Cls. Right: composite desirability (0-1) balancing high titer with acceptable VCD and viability. Red dashed lines mark targets/constraints.

Sequence-verified plasmids were used for transient transfection of CHO cells in 96 half–deep-well plates, performed as two independent biological replicates for each LC/HC promoter condition and antibody (B, C, E). To assess transfection efficiency, three control wells were transfected with a GFP expression plasmid. Flow-cytometry measurement of GFP indicated 91% GFP-positive cells (n = 3 wells; Figure 2D), indicating uniform and efficient delivery of DNA across the plate.

### Optimal heavy to light chain ratio for transient monoclonal antibody production

A custom D-optimal design was employed to identify the optimal balance between LC and HC promoters, with strengths tested at low (5 RPU), medium (40 RPU), and high (100 RPU) levels. The design included nine randomized LC/HC promoter combinations in duplicate. Transfections were performed in 96-well half-deep plates, and antibody titer (mg/L), viable cell density (cells/mL), and viability (%) were measured 72 h post-transfection.

For antibody B, the highest titer was achieved with a high LC promoter combined with medium or high HC promoter strengths (LC100–HC40, LC100–HC100). However, pairing high LC and high HC (LC100–HC100) decreased cell viability and viable cell density, likely due to overexpression stress. Combinations using a medium LC promoter with medium or high HC (LC40–HC40, LC40–HC100) preserved viability and cell density but yielded intermediate titers. By contrast, any condition incorporating a low-strength promoter for either chain (LC40–HC5, LC100–HC5, LC5–HC100, LC5–HC40, LC5–HC5) produced the lowest titers (Figure 2A–C). The JMP profiler (Figure 2E) visualized these response surfaces: predicted titer rises with LC strength and peaks at LC100 with HC40–100, whereas both viable cell density and viability drop specifically at LC100–HC100. The profiler’s confidence intervals and prediction traces indicate stronger sensitivity to LC than HC for titer, with a ridge of high productivity at high LC and non-low HC. A composite desirability function that prioritizes high titer while maintaining acceptable cell density and viability identifies LC100–HC40 as the optimal operating point. Together, measurements and model converge on a high-LC and medium-HC configuration as the best balance of productivity and cell health for mAb B.

**FIGURE 3:**
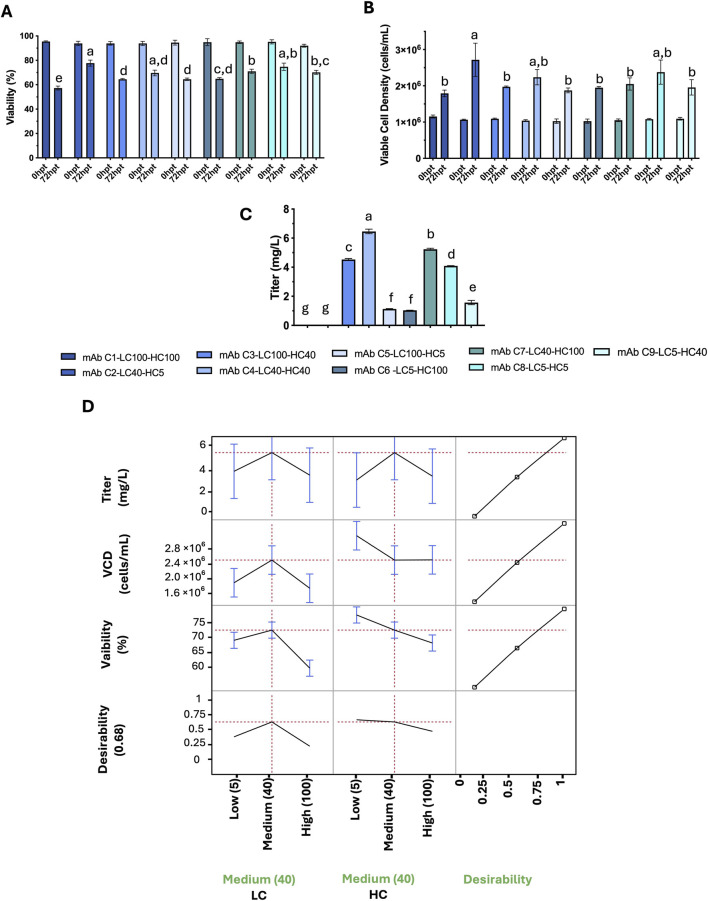
mAb C: effects of LC/HC promoter strength on titer, cell density, and viability post-transfection (72 hpt), and model profiling. **(A)** Viability (6), **(B)** viable cell density (VCD; cells/mL), and **(C)** titer (mg/L) for all LC/HC combinations. Promoter levels: 5, 40, and 100 RPU for both LC and HC. Bars show mean = SD (n = 2). Statistical analysis was based on one-way ANOVA with Tukey’s HSD; different letters indicate p < 0.05. **(D)** JMP profiler for least-squares models of titer, VCD, and viability versus LC/HC promoter strength. Left: predicted means with 95% Cls. Right: composite desirability (0-1) balancing high titer with acceptable VCD and viability. Red dashed lines mark targets/constraints.

For antibody C, the highest titer was achieved with a medium LC promoter combined with a medium HC promoter (LC40–HC40), with cell density and viability remaining acceptable. The combination of a medium LC with a high HC promoter (LC40–HC100) yielded the second-highest titer without adverse effects on cell density or viability, and the high LC with medium HC condition (LC100–HC40) produced the third-highest titer. Notably, using high LC and high HC promoters together (LC100–HC100) diminished antibody C production and reduced cell viability. Similar to antibody B, incorporating a lowest-strength promoter for either chain adversely affected titer (LC40–HC5, LC5–HC100, LC5–HC40). Interestingly, the lowest-strength combination for both chains (LC5–HC5) resulted in the fourth-highest titer for antibody C (Figure 3A-C). The JMP profiler mirrors these trends. Predicted titer peaks at LC40 with HC40–100, indicating greater sensitivity to LC around the medium setting and a smaller, positive contribution from higher HC. VCD and viability are broadly maintained across most settings but decline at LC100–HC100. When the three responses are combined in a composite desirability (0–1) that prioritizes titer while constraining VCD and viability, the optimum lies at LC40–HC40, with LC40–HC100 as a close alternative. Thus, experimental data and model predictions converge on medium LC with medium–high HC as the preferred operating region for mAb C (Figure 3D).

**FIGURE 4:**
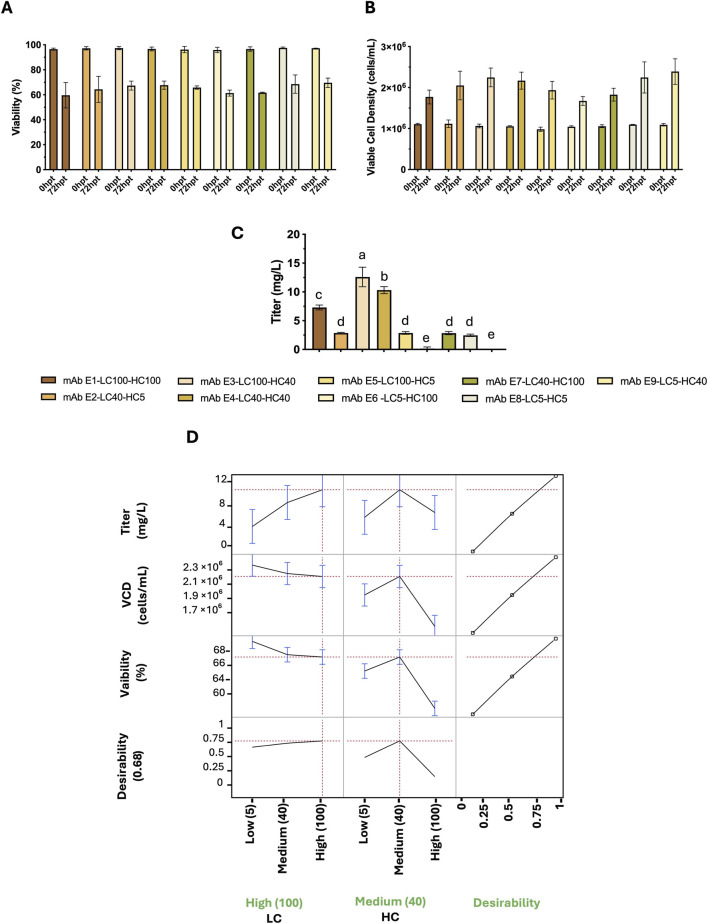
mAb E: effects of LC/HC promoter strength on titer, cell density, and viability post-transfection (72 hpt), and model profiling. **(A)** Viability (%), **(B)** viable cell density (VCD; cells/mL), and **(C)** titer (mg/L) for all LC/HC combinations. Promoter levels: 5, 40, and 100 RPU for both LC and HC. Bars show mean ± SD (n = 2). Statistical analysis was based on one-way ANOVA with Tukey’s HSD; different letters indicate p < 0.05. **(D)** JMP profiler for least- squares models of titer, VCD, and viability versus LC/HC promoter strength. Left: predicted means with 95% CIs. Right: composite desirability (0-1) balancing high titer with acceptable VCD and viability. Red dashed lines mark targets/constraints.

Finally, for antibody E, the highest titer was achieved with a high LC promoter combined with a medium HC promoter (LC100–HC40), while cell density and viability remained unchanged. The next-highest titer was observed with the medium LC and medium HC combination (LC40–HC40). The high-LC/high-HC condition (LC100–HC100) yielded a lower titer. Similar to the other antibodies, incorporating a low-strength promoter for either chain reduced titer to the lowest levels (LC40–HC5, LC100–HC5, LC5–HC100, LC5–HC40, LC5–HC5). Interestingly, cell viability and viable cell density did not differ significantly across LC/HC promoter combinations (Figure 4A-C). The JMP profiler (Figure 4D). Aligns with these measurements. Predicted titer increases with LC strength and is maximized at LC100 with HC40 (and declines when HC is pushed to 100). Predicted VCD and viability show minimal sensitivity to LC/HC over the tested range, consistent with the experimental means. Because the desirability function prioritizes high titer while constraining VCD and viability within acceptable ranges, it selects LC100–HC40 as the optimal operating point, with LC40–HC40 as a lower-titer but robust alternative. Collectively, the data and model point to high LC with medium HC as the preferred balance of productivity and cell health for mAb E.

## Discussion

This work presents a microscale, DoE-guided platform that systematically optimizes the expression balance of antibody heavy and light chains through promoter tuning in CHO cells, effectively integrating construct design and bioprocess development within a single, high-throughput workflow.

Over the past decade, the number of non-natural mAbs has increased exponentially, driven by the emergence of bispecific (BsAbs) and multispecific antibodies (Brinkmann and Kontermann, 2017; Crescioli et al., 2024). This diversification in primary sequences has put the CHO biomanufacturing platform to the test. Changes in the antibody sequence can negatively impact production, often resulting in suboptimal cell growth, low specific productivity, and reduced product quality (Li et al., 2022; Yang et al., 2022; Rahimi et al., 2025). The factors contributing to lower yields of these difficult-to-express (DTE) antibodies include folding inefficiencies, mismatched assembly, intracellular accumulation, and formation of chain aggregates, among others (Papadaki et al., 2025; Szkodny and Lee, 2025). Among the challenges that can be addressed through cell line engineering, and a key strategy for many antibodies involves optimizing the heavy chain to light chain ratio to improve expression and overall production efficiency (Pybus et al., 2014; Zhang et al., 2022).

Natural mAbs are composed of equimolar heavy and light chains. However, in CHO cells, this ratio is often non-optimal, particularly when expressing alternative or artificially designed sequences (Schlatter et al., 2005; Zhang et al., 2022). Traditionally, recombinant mAb production relies on expressing the heavy and light chains from separate vectors, making precise control of their ratio challenging. Imbalances can lead to an excess of either chain, promoting intracellular aggregation, misfolding, ER stress, and activation of the unfolded protein response (UPR), which may result in degradation or even apoptosis (Chung et al., 2018; Mathias et al., 2020). Such imbalances reduce overall yield and product quality, as unassembled aggregates or dimers may be secreted alongside correctly assembled antibodies.

Our findings confirm that the optimal LC/HC promoter ratio is antibody dependent, aligning well with previous reports highlighting the importance of balancing heavy and light chain expression for efficient mAb assembly and secretion. In our study, the best-performing configurations, high LC with medium HC expression for two antibodies and medium LC with medium HC for a third, reflect this balance between productivity and cellular health. The decrease in viable cell density observed at the highest expression levels (LC100–HC100) is consistent with previous observations that overly strong promoters can overload the folding and secretory machinery, compromising culture performance despite higher titers. Conversely, low promoter strengths for either chain consistently resulted in poor titers, reinforcing the need for a sufficient expression threshold to enable efficient assembly. Overall, our results corroborate literature trends while emphasizing that the optimal HC/LC ratio must be empirically determined for each antibody, as sequence-specific folding kinetics and assembly efficiencies shape the ideal expression balance for maximizing yield and maintaining cell viability.

Our high-throughput approach allows fine-tuning of this ratio for each antibody, improving yield and reproducibility across the tested sequences. The systematic generation of quantitative data on promoter performance and expression balance supports predictive modelling and rational design of expression constructs. By integrating rapid screening with data analysis, this method facilitates fast iteration and standardization of construct optimization for new antibody candidates, thereby accelerating development timelines in biotherapeutic pipelines.

Our method uses 96-well format from cloning to CHO culture and transfection to titer reading. Building on previous work (Hansen et al., 2015) which demonstrated the feasibility of small-scale suspension CHO cultures in 96-well plates, we leveraged this framework to develop a high-throughput platform compatible with our expression optimization workflow. In this study, we expanded the 96-well format to include high-throughput cloning and systematic evaluation of heavy and light chain promoter combinations, obtaining a transfection efficiency of 91%. By coupling this microscale screening with DoE analyses, we enable rapid, quantitative assessment of key responses while significantly reducing experimental time and resource requirements. This integrated approach maintains scalability relevance for suspension systems while allowing early data-driven construct optimization.

For the D optimal custom-designed DoE approach, we analyzed three different antibody sequences, evaluating two categorical factors (HC and LC) at three expression levels (5, 40, and 100 RPU) and three response variables: viable cell density (cells/mL), viability (%) and titer (mg/L). This design provides versatility and can be adapted to optimize these parameters under various production scenarios, as well as extended to other biological systems. Beyond the three analyzed responses, the approach can incorporate any quantifiable parameter relevant to process optimization. Our high-throughput screening platform enables the inclusion of additional responses, such as maximum growth rate, glucose consumption, lactate production, or other key metabolites, within the DoE framework. Furthermore, the design can be modified to evaluate continuous factors, including sequence length, inducer concentration in inducible systems, or harvest time. Overall, this method combines high-throughput transfection in 96-well plates with DOE-based analysis, offering a flexible and scalable strategy for systematic bioprocess development.

### Translatability to stable cell lines

**FIGURE 5:**
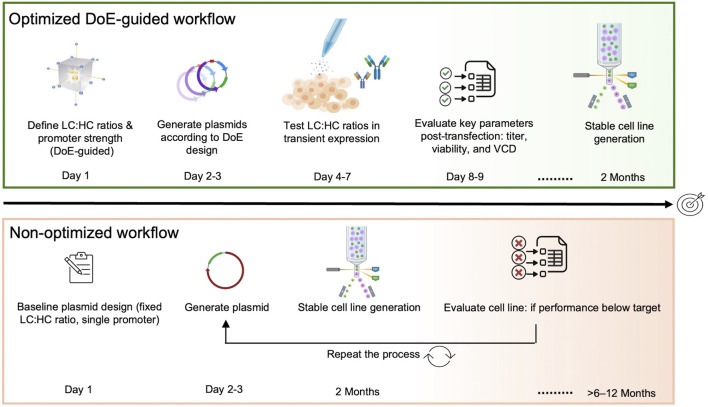
Comparison of optimized and conventional cell line development workflows. The optimized workflow (top) employs a fully high-throughput, DoE-guided expression design strategy in which LC and HC expression levels are systematically tuned through promoter strength selection. High- throughput transient screening enables rapid evaluation of expression performance across multiple construct combinations and early identification of optimal vector designs. This integrated approach allows completion of stable cell line generation within approximately 2 months. In contrast, the conventional non-optimized workflow (bottom) relies on baseline vector designs with fixed promoter configurations and expression parameters. When suboptimal performance is identified only after stable cell line generation, iterative vector redesign, recloning, and repeated cell line development cycles are required, resulting in substantially extended timelines (>6-12 months).

A central objective of this study was to establish a high-throughput screening framework that enables efficient translation of early construct-level decisions into stable CHO cell line development (Figure 5). While transient transfection systems provide a rapid and scalable platform for comparing large numbers of design variants, their ultimate value lies in informing construct choices that perform robustly after genomic integration.

In transient expression, the LC/HC ratio can be readily modulated by adjusting plasmid ratios during transfection. However, such an approach is not directly applicable to stable cell line generation, where expression levels are dictated by the integrated construct architecture. Consequently, controlling LC/HC balance in a stable context requires systematic evaluation of design elements embedded within the expression vector itself. In this work, we therefore focused on promoter strength combinations as a tunable and translatable means of modulating LC and HC expression at the construct level.

To further reduce variability associated with genomic integration, we can employ a targeted-integration CHO system, enabling control over integration site and copy number. This strategy minimizes positional effects and clonal variability, thereby facilitating a more direct translation of promoter-driven expression differences observed during high-throughput screening to stable production settings (Rahimi et al., 2025).

We acknowledge that a limitation of the presented high-throughput method is that optimization was primarily guided by quantitative process-level parameters (titer, VCD, and viability), and that the small culture volumes used in the screening workflow do not readily permit comprehensive assessment of product quality attributes. To address this, we extended the workflow beyond the screening stage by generating a representative stable cell line based on the optimal promoter combination identified for antibody C. Analysis of antibodies produced in batch culture demonstrated improved product quality, with no detectable free LC or HC monomers or dimers compared to a non-optimized control (Supplementary Figure S6). These results support the translatability of the screening framework to stable cell line development and demonstrate its value as an early-stage, systematic tool for identifying promoter combinations that enable both efficient production and correct antibody assembly. Nevertheless, we acknowledge that broader validation across multiple antibodies, integration events, and long-term cultures would be required to fully generalize these findings. Subsequent optimization of additional vector elements and extended quality characterization can then also be pursued as part of downstream cell line development.

## Data Availability

The original contributions presented in the study are included in the article/Supplementary Material, further inquiries can be directed to the corresponding author.
