# Supplementary material for: High-throughput optimization of antibody production in CHO cells by tuning heavy- and light-chain promoter strength
Source: Front Bioeng Biotechnol. 2026 Jan 9;13:1747473. doi: 10.3389/fbioe.2025.1747473 (PMC12827772; doi:10.3389/fbioe.2025.1747473)
Supplement: Supplementary file 1 [file DataSheet1.pdf]

Supplementary Figure S1: Plasmid maps of (1A-C) backbone and (2.D-F) stuffer plasmids with three promoter strength used for final plasmid generation.

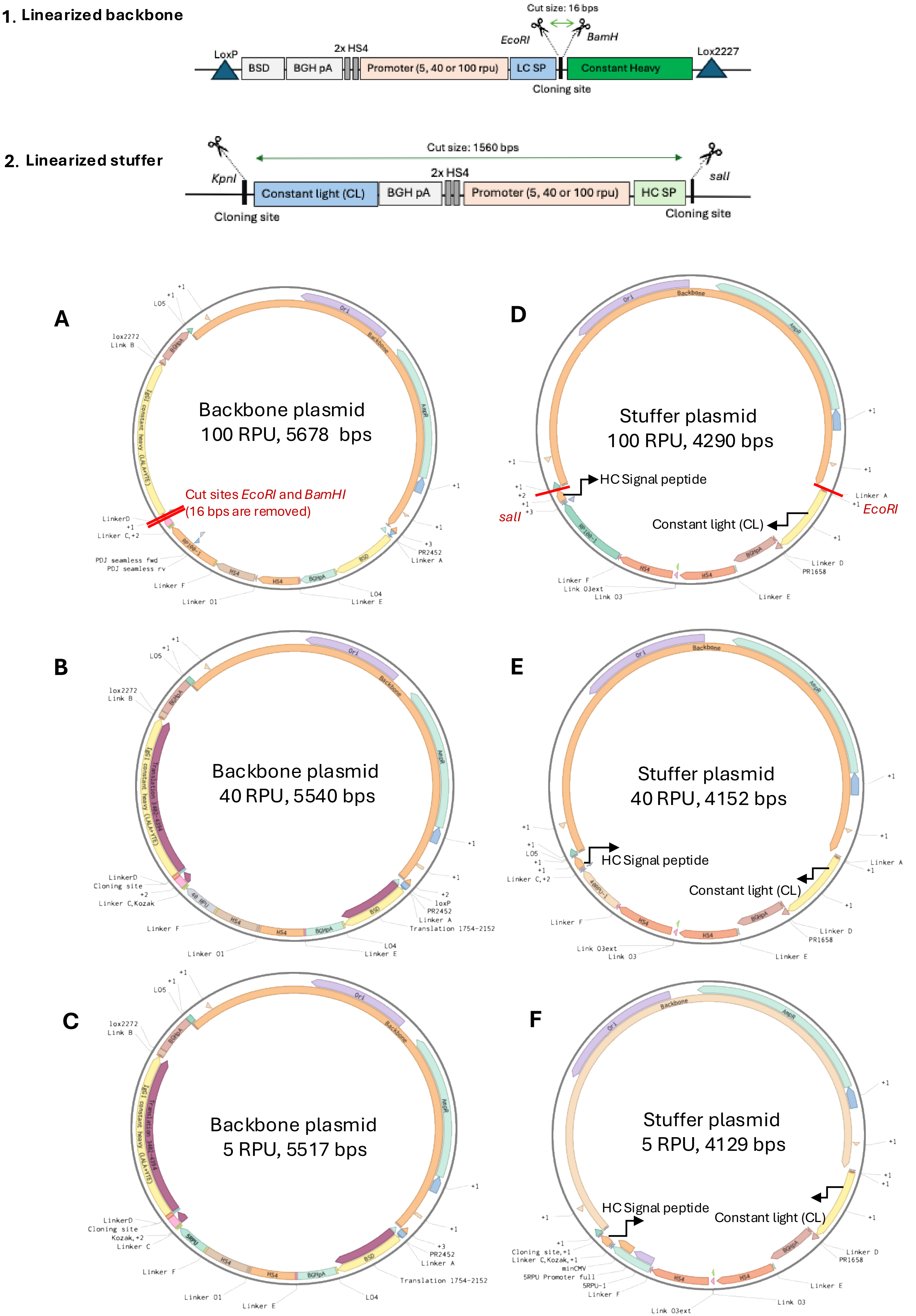

Supplementary Figure S2: Agarose gel electrophoresis of backbone and stuffer plasmids following restriction enzyme digestion. (A) Backbone plasmids digested with *BamHI* and *EcoRI*. The expected fragment sizes were: 100 RPU, 5662 bp and 16 bp; 40 RPU, 5524 bp and 16 bp; and 5 RPU, 5501 bp and 16 bp. (B) Stuffer plasmids digested with *SalI* and *KpnI*. The expected fragment sizes were: stuffer-100 RPU, 1560 bp and 2730 bp; stuffer-40 RPU, 1422 bp and 2730 bp; and stuffer-5 RPU, 1399 bp and 2730 bp. The fragments were separated on a 1% (w/v) agarose gel prepared in 1× TAE buffer, with a 1 kb DNA ladder as a molecular size marker and visualized using SYBR Safe DNA Gel Stain.

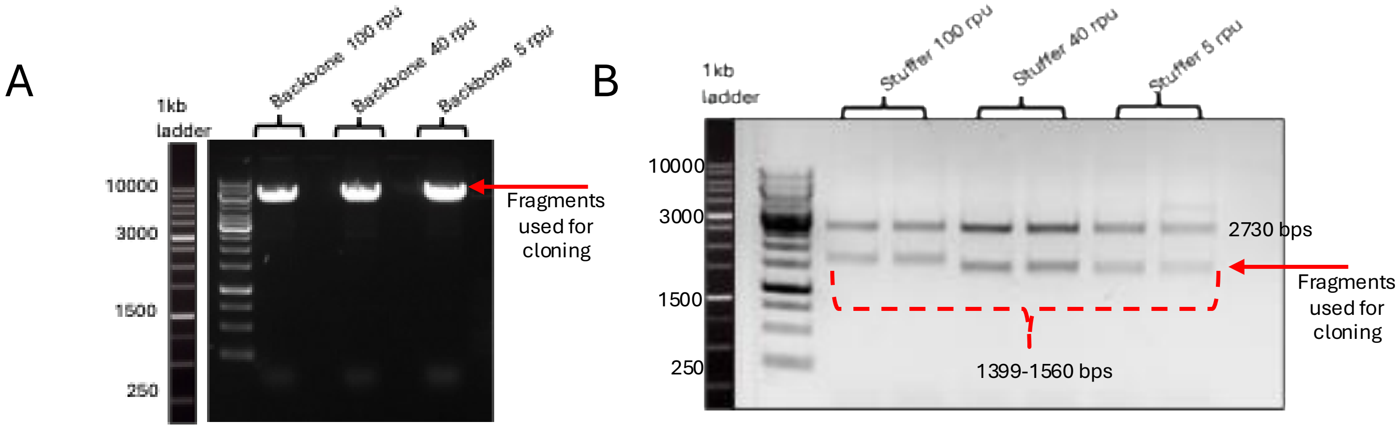

Supplementary Figure S3: Agarose gel electrophoresis of PCR-amplified variable regions (VL and VH) of antibodies B, C, and E. The expected fragment sizes are as follows: mAb B — VL: 333 bp, VH: 363 bp; mAb C VL: 336 bp, VH: 393 bp; mAb E — VL: 327 bp, VH: 351 bp. PCR products were separated on a 1% (w/v) agarose gel prepared in 1× TAE buffer, with a 1 kb DNA ladder as a molecular size marker and visualized using SYBR Safe DNA Gel Stain. VL=variable light. VH= variable heavy

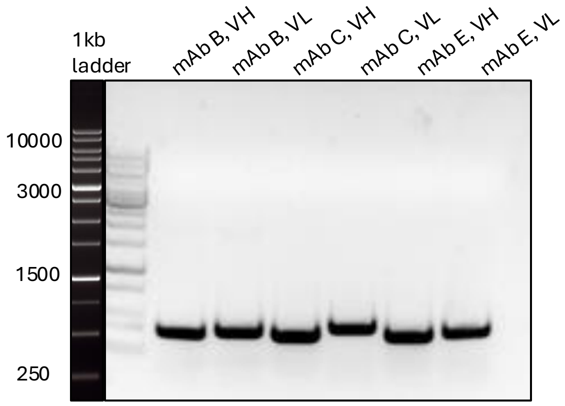

Supplementary Figure S4: Flow-cytometry gating and GFP expression. Events were gated for Cells and then Single cells (FSC/SSC and doublet exclusion). GFP positivity was defined on FITC-A vs FSC-A. Histograms show the fluorescence distributions for the gated single-cell population. Transfection was done in three biological replicates.

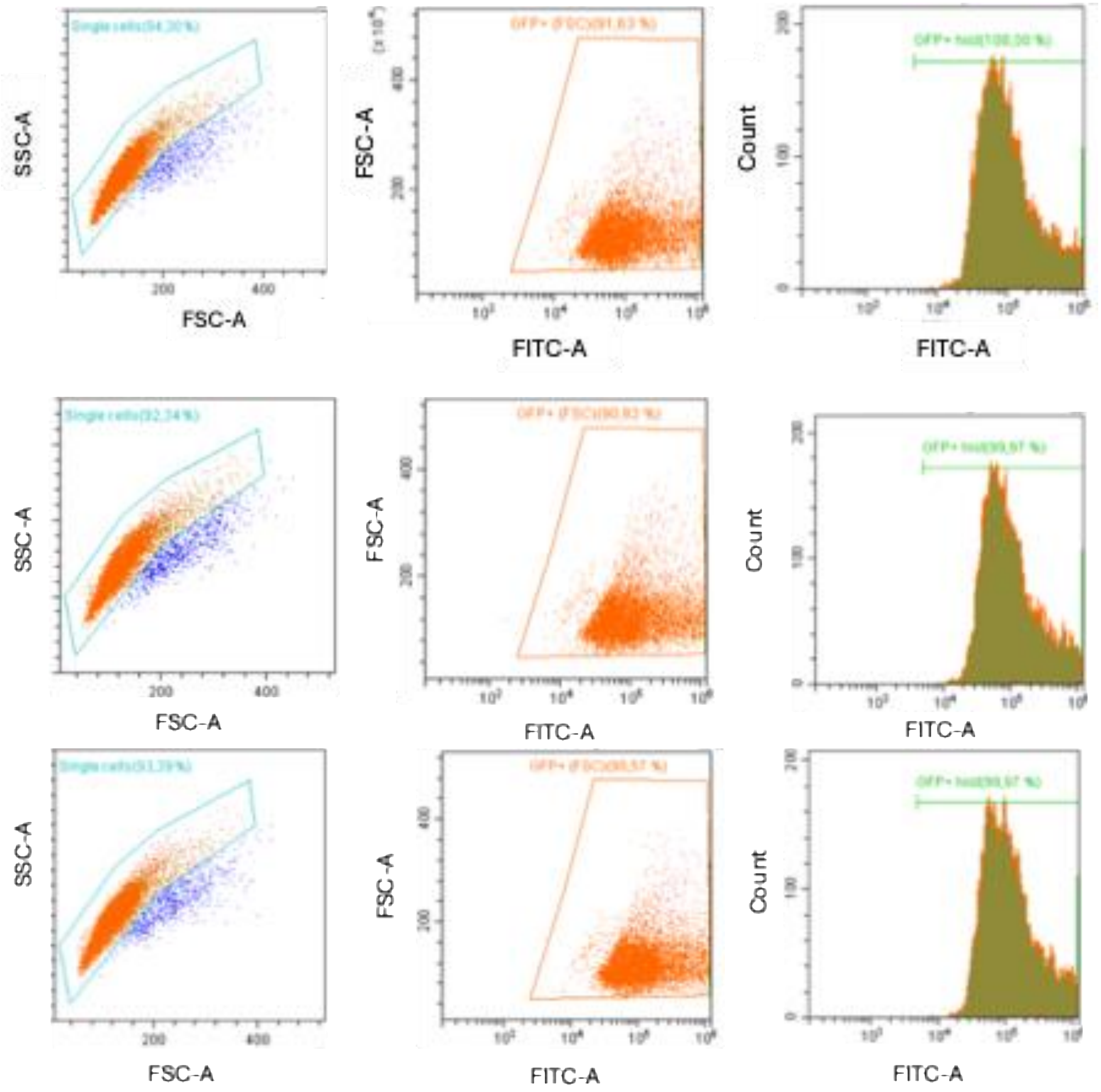

Supplementary Figure S5: Agarose gel electrophoresis of colony PCR products confirming the correct assembly of final antibody coding plasmids (with different combination of light and heavy chain promoters). The forward primer anneals to the stuffer region (CL), and the reverse primer to the backbone region (CH). The expected amplicon size is 2130 bp. PCR products were separated on a 1% (w/v) agarose gel prepared in 1× TAE buffer, with a 1 kb DNA ladder as a molecular size marker and visualized using SYBR Safe DNA Gel Stain.

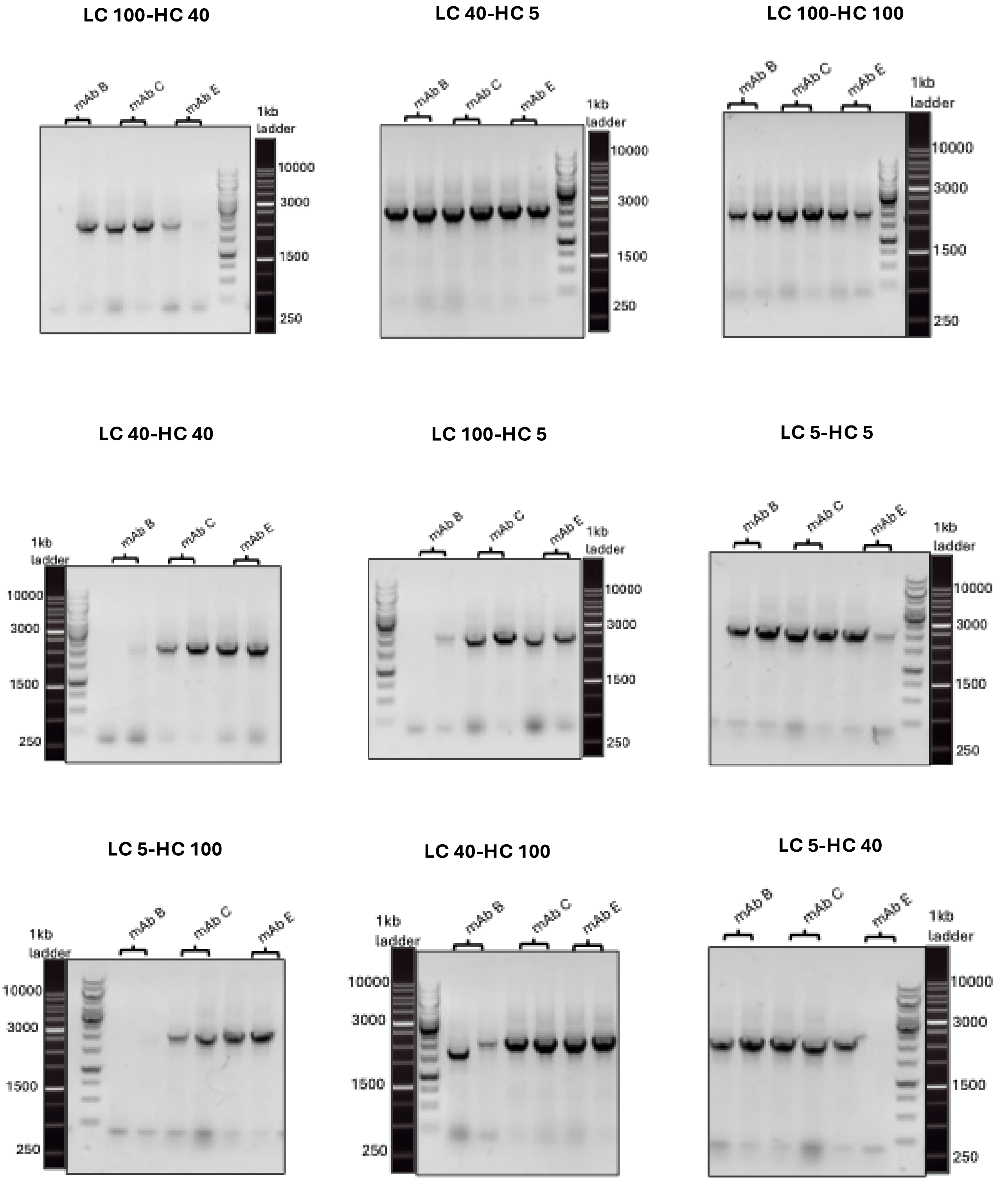

Supplementary Figure S6: Stable cell line generation and quality analysis for antibody C. A) Western Blot with an antibody binding to the kappa constant region of the light chain. Samples come from a batch culture showing results from day 1 to 7. The antibody binds all full IgGs at ~150 kDa. The observed bands at ~50 kDa and ~25 kDa are assumed to represent free light chain monomers (LC) and dimers (LC_2). B) Western Blot with an antibody binding to the Fc IgG1 region of the heavy chain. Results showed only for day 7 of batch culture. The observed prominent band at ~100 kDa is assumed to be heavy chain dimers (HC_2). C) Full gels images.

Method for stable cell line generation and batch culture: Cell lines were generating transfecting 12-G12-002 CHO master cell lines previously developed by the group containing a landing pad at the T9-NW-003614758.1 locus, which facilitates the integration of antibody genes through RMCE. The MCL was cultured in CD CHO medium supplemented with 8 mM L-Glutamine and maintained in 125 mL Erlenmeyer flasks with vent caps, incubated at 37 °C, 5% CO2, and agitated at 130 rpm. Passaging occurred every 2-3 days, with cell growth and viability monitored using the NucleoCounter NC-200.

MCLs at a concentration of 1 × 106 cells/mL were transfected with Cre recombinase and antibody-encoding plasmids at a ratio of 1:3 (w:w) in 6-well plates using FreeStyle MAX transfection reagent according to the manufacturer's recommendations. Single cell were sorted by fluorescence-activated cell sorting (FACS) isolating mCherry-negative cells in 384-well plates in 30 µL of CD CHO medium supplemented with 8 mM L-glutamine, 1.5% HEPES and 1% Antibiotic-Antimycotic. 14 days after FACS, clones were transferred to flat-bottom 96-well plates containing CD CHO medium supplemented with 8 mM L-glutamine and 1% Antibiotic-Antimycotic. Targeted integration was verified by junction PCR, followed by copy-number analysis via qRT-PCR. Verified clones were expanded and cryopreserved at −180 °C.

For the **batch culture**, cells were seeded at a density of 3 x 10^5 cells/mL in 30 mL of CD CHO medium supplemented with 8 mM L-Glutamine and samples were taken for antibody titer assessment every 24h.

More details about cell line generation can be found in M. Rahimi et al. 2025 (DOI: 10.1021/acssynbio.5c00861)

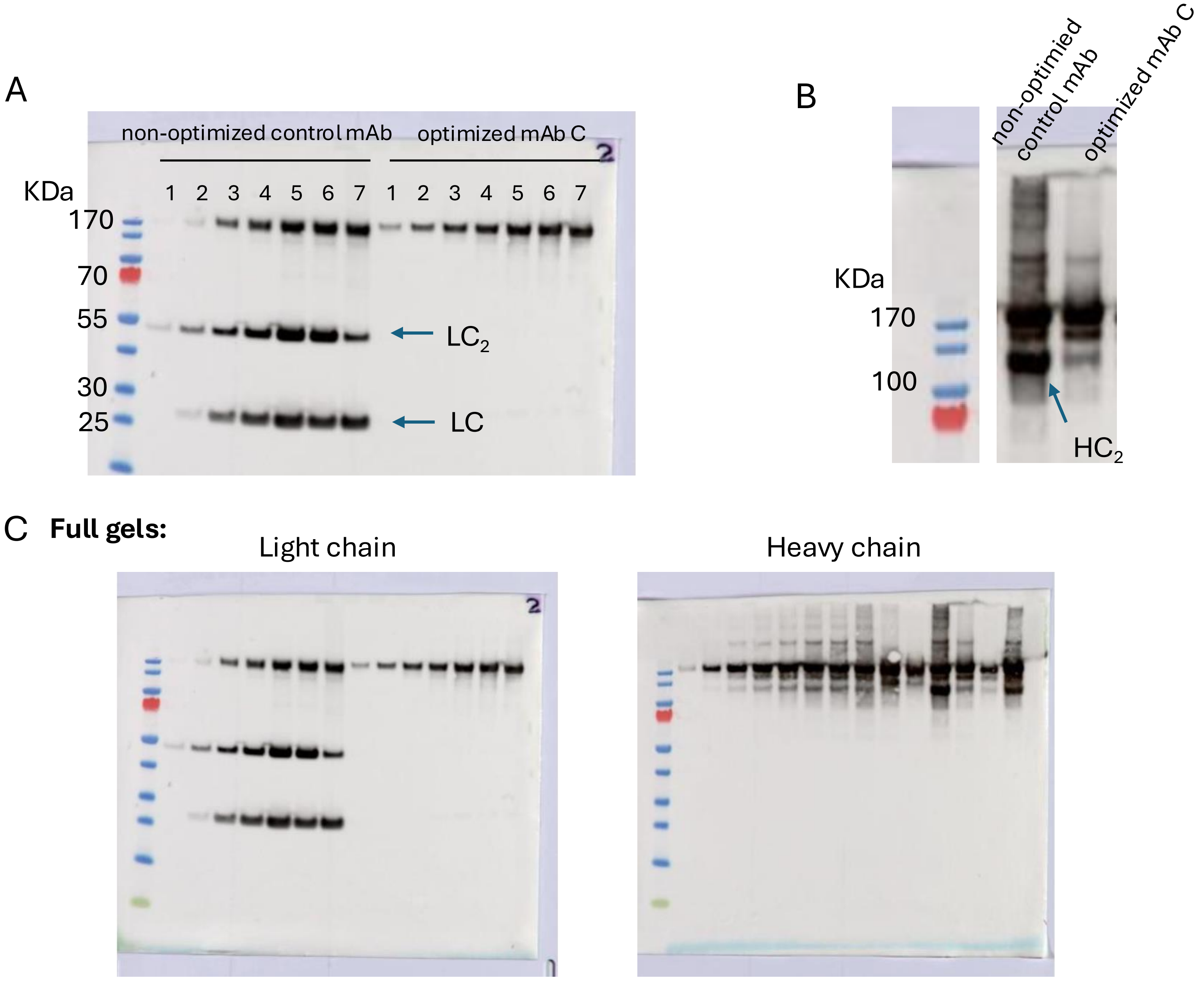

Supplementary Table S1. Sequences of variable regions for antibodies B, C, and E, and the constant light (CL) and constant heavy (CH) chains used as the constant-region backbone.

Antibody	Sequences (5’-> 3’)
Mab B-VL	AACTTCATGCTGACCCAGCCTCACAGCCTGTCTGAGTCTCCAGGCAAGACCGTGACAATCAGCTGCACCAGATCCTC<br>CGGCTCTATCGCCAACAACTAGTGCAGTGGTATCAGCAGCGGCCTGGCTCCTCTCCTACCACCGTGATCTACGAGGA<br>CAACCAGCGGCCTTCTGCCGTGCCTGATAGATTTTCCGGCTCCATCGACTCCTCCAGCAACTCCGCTTCTCTGACCAT<br>CAGCGGCCTCAAGCCGAGGACGAGGCCGATTACTACTGCCAGTCCTACGACTCCTCTAACCACTGGGTTTTCGGCG<br>GAGGCACCAAGCTGACAGTTCTG
Mab B-VH	GAAGTGCAGCTGGTTCAGTCTGGCGCCGAAGTGAAGAAGCCTGGCGAGTCCCTGCGGATCTCCTGTAAAGGCTCCG<br>GCTACTCCTTTACCTCCTACTGGATCAGCTGGGTCCGACAGATGCCTGGCAAAGGCCTGGAATGGATGGGCAGAATC<br>GACCCCTCCGACTCCTATACCAACTACTCCCCTAGCTTCCAGGGCCACGTGACCATCTCTGCCGACAAGTCTATCTCC<br>ACCGCCTACCTGCAGTGGTCCTCTCTGAAGGCCTCTGACACCGCCATGTACTACTGTGCCAGACAGGGCGACTACTA<br>CGGCTCCGGCAGATATCCTCATTGGGGCCAGGGAACCCTGGTCACCGTGTCCTCT
Mab C-VL	AACTTCATGCTGACCCAGCCTCACAGCGTCAGCGAATCTCCCGGAAAAACCGTCACCTTCTCCTGTACCAGATCCTCT<br>GGCAGAATCGTGTCCGACTATGTGCACTGGTACCAGCAGCGGCCTGGCTCTGCTCCTACAACAGTGATCTACGAGGA<br>CAACCAACGCCCAAGCGGCGTGCCTGATCGGTTTTCTGGCTCCATCGACTCCAGCTCCAACTCCGCCTCTCTGACCA<br>TCTCCGGCCTGAAGACCGAGGATGAGGCCGACTACTACTGCCAGTCCTACGACTCTTCTAATGCCTACGTGGTGTTCG<br>GCGGAGGCACCAAGGTGACCGTGCTG
Mab C-VH	CAGGTGCAGCTGGTGCAGTCTGGCGCCGAGGTGAAGAAGCCTGGCTCTAGCGTGAAAGTGTCTTGCAAGGCCAGCG<br>GCGGAACATTTTCCTCCTACGCCATCTCCTGGGTGCGGCAGGCTCCTGGACAAGGCCTGGAGTGGATGGGCGGCAT<br>CATCCCCATCTTCGGCACCGCCAACTACGCTCAGAAGTTCCAGGGCAGAGTGACCATCACCGCCGACGAGTCCACC<br>TCTACCGCCTACATGGAACTGCGGAGCCTGAGATCCGATGATACCGCTGTGTACTACTGTGCTCGCGACAACCTGGGC<br>TACTGCTCCGGCGGCTCCTGCTACTCCGACTACTACTACTATTACATGGACGTGTGGGGCCAGGGAACCCTGGTCACA<br>GTGTCCTCT
Mab E-VL	GACATCGTGATGACCCAGTCTCCTGGCACCCTGAGCCTGTCTCCAGGCGAGCGGGCCACACTGTCCTGCAGAGCCT<br>CTCAGTCCGTGTCCAGCTCCTACTCCGCCTGGTACCAGCAGAAACCCGGCCAGGCCCCTCGGCTGCTGATCTATGG<br>CGCTAGCTCTCGGGCTACAGGCATCCCCGATAGATTCTCCGGATCTGGCTCTGGAACCGACTTCACCCTGACCATCTC<br>TAGACTGGAACCTGAGGACTTTGCCGTGTACTACTGTCAACAGTACGGCTCCTCCCCTCCTTACACCTTCGGCCCTGG<br>CACCAAGGTCGAGATCAAG
Mab E-VH	GAGGTCCAGCTGGTGCAGTCCGGCGCCGAGGTGAAGAAGCCTGCTGCTTCCGTGAAAGTGTCCTGCAAGGCCTCTG<br>GCTACACCTTCACCGGCTACTACATGCACTGGGTGCGCCAGGCCCCTGGCCAGGGCCTGGAATGGATGGGCATCAT<br>CAACCCCAGCGGCGGCTCCACATCCTACGCCCAGAAGTTCCAGGGCAGAGTGACCATGACCAGAGATACCTCCAC<br>ATCTACCGTGTACATGGAACTGTCTTCTCTGAGATCTGAGGACACCGCTGTGTACTATTGTGCCCGGGAGTGGGGATAC<br>GGCATGGACGTGTGGGGCCAAGGAACCACCGTGACAGTCAGCAGC
Constant light (CL)	CGGACCGTGGCCGCTCCCTCCGTGTTCATCTTCCCACCTTCCGACGAGCAGCTGAAGTCCGGCACCGCTTCTGTCG<br>TGTGCCTGCTGAACAACTTCTACCCCCGCGAGGCCAAGGTGCAGTGGAAGGTGGACAACGCCCTGCAGTCCGGCA<br>ACTCCCAGGAATCCGTGACCGAGCAGGACTCCAAGGACAGCACCTACAGCCTGTCCTCCACCCTGACCCTGTCCAA<br>GGCCGACTACGAGAAGCACAAGGTGTACGCCTGCGAAGTGACCCACCAGGGCCTGTCTAGCCCCGTGACCAAGTCT<br>TTCAACCGGGGCGAGTGCTGA
Constant Heavy (CH)	GCTTCTACCAAGGGCCCCTCCGTGTTCCCTCTGGCCCCTTCCAGCAAGTCTACCTCTGGCGGCACAGCCGCTCTGG<br>GCTGCCTCGTGAAGGACTACTTCCCCGAGCCCGTGACAGTGTCCTGGAACTCTGGCGCTCTGACCAGCGGAGTGCA<br>CACCTTCCCTGCTGTGCTGCAGTCCTCCGGCCTGTACTCCCTGTCCAGCGTCGTGACTGTGCCCTCCAGCTCTCTGG<br>GCACCCAGACCTACATCTGCAACGTGAACCACAAGCCCTCCAACACCAAGGTGGACAAGAAGGTGGAACCCAAGTC<br>CTGCGACAAGACCCACACCTGTCCCCCTTGTCCTGCCCCTGAAGCCGCCGGCGGACCCAGCGTGTTCCTGTTCCC<br>CCCAAAGCCCAAGGATACCCTGTACATCACCCGGGAGCCCGAAGTGACCTGCGTGGTGGTGGATGTGTCCCACGAG<br>GACCCTGAAGTGAAGTTCAATTGGTACGTGGACGGCGTGGAAGTGCACAACGCCAAGACCAAGCCTAGAGAGGAAC<br>AGTACAACTCCACCTACCGGGTGGTGTCCGTGCTGACCGTGCTGCACCAGGATTGGCTGAACGGCAAAGAGTACAAG<br>TGCAAGGTGTCCAACAAGGCCCTGCCTGCCCCCATCGAAAAGACCATCTCCAAGGCCAAGGGCCAGCCCCGGGAA<br>CCCCAGGTGTACACACTGCCCCCTAGCAGGGACGAGCTGACCAAGAACCAGGTGTCCCTGACATGCCTCGTGAAAG<br>GCTTCTACCCCTCCGATATCGCCGTGGAATGGGAGTCCAACGGCCAGCCTGAGAACAACTACAAGACCACCCCCCC<br>TGTGCTGGACTCCGACGGCTCATTCTTCCTGTACAGCAAGCTGACAGTGGACAAGTCCCGGTGGCAGCAGGGCAAC<br>GTGTTCTCCTGCTCCGTGATGCACGAGGCCCTGCACAACCACTATACCCAGAAGTCCCTGTCCCTGAGCCCCGGCAA<br>GTGA

Supplementary Table S2. Promoter sequences (5, 40, 100 RPUs) used for antibodies B, C, and E.

Promoters	Sequences (5'-> 3')
**Promoter 5 RPU-1- minCMV**	TATAGGAAGGTCTTACCGGAAGTTCCTTAGCTGATAGTATACCAGATTTTTGCG CAATTCTAACTGATCATCTAACGACCTATTACCGGAAGTTAGTATGTGTACAAAAG GTCTATATAAGCAGAGCTCGTTTAGTGAACCGTCAGATCGCCTAGATACGCCAT CCACGCTGTTTTGACCTCCATAGAAGAC
**Promoter 40 RPU-1+ minCMV**	TTTTGCGCAATTTATAGGTGGGGCGGGGAAAGGTCATGACACAGCAATCAGAT TTGCTTGCGTGAGAAGAAGTATGTTACCGGAAGTTG ACCTATGGGACTTTCCATCTAACATGACACAGCAATTGTACAAAAGGTCTATATA AGCAGAGCTCGTTTAGTGAACCGTCAGATCGCCTAGATACGCCATCCACGCTG TTTTGACCTCCATAGAAGAC
**Promoter 100 RPU-2+minCMV**	TGGGACTTTCCACCTTAGATGACACAGCAATCAGATTTGCTTGCGTGAGAAGAT ATAGGATGACACAGCAATCTAGACTGGGACTTTCCACTGATATTTTGCGCAATTG ACCTAATGACACAGCAATAGTATGTGGGGCGGGGATCTAACTGGGACTTTCCA AAGGTCTTACCGGAAGTTGTTAGAATGACACAGCAATGGATTCATATCCTGGGA CTTTCCAGTATACTGCTTGCGTGAGAAGATGATCATGGGACTTTCCATGTACAAA AGGTCTATATAAGCAGAGCTCGTTTAGTGAACCGTCAGATCGCCTAGATACGCC ATCCACGCTGTTTTGACCTCCATAGAAGAC

Supplementary Table S3. List of primers used for amplification of stuffer and backbone plasmids, as well as antibody variable regions

Primer name	Sequences (5'-> 3')
BBPromoter_5RPU-1. FW	CTGAGTGCACGAACGTGGATTATAGGAAGGTCTTACCG
BBPromoter_5_RPU-1. Rev	CCATGGTGGCGATGACGTCTGTCTTCTATGGAGGTCAAAAC
BBPromoter_40RPU-1. FW	CTGAGTGCACGAACGTGGATTTTTGCGCAATTTATAGGTG
BBPromoter_40RPU-1.REV	CCATGGTGGCGATGACGTCTGTCTTCTATGGAGGTCAAAAC
BBPromoter_100RPU-1. FW	ACAACTCCGGTAAGACCTTTGGAA
BBPromoter_100RPU-1.REV	AAGTTGTAGAATGACACAGCAATG
Stuffer- universal -Fw	AGACGTCATcgccaccATGGGCTG
Stuffer- universal -Rev	ATCCACGTTCGTGCACTCAGCCTAAAG
VH_FW_ universal	gttgccacccgggtcctgtctGAGGTGCAGCTGGTGCAGTCC
VH_Rev_ universal	ggaggggcccttggtagaagcAGAGGACACGGTGACCAGGGT
VH_Rev_E	ggaggggcccttggtagaagcGCTGCTGACTGTCACGGTGGT
VL_FW_ universal	gccagcgtgatcatgtcccggggcGACTTCGTGCTGACCCAGTCT
VL_Rev_ universal	cacggagggagcggccacggtccgCTTGATCTCGACCTTGGTGCC
Colony PCR Fw	TGTTCATCTTCCCACCTTCC
Colony PCR rev	GACTTGGGTTCCACCTTCTT

Supplementary Table S4. Design matrix and observed responses for mAb B (Custom D-optimal DoE). Eighteen runs cover the nine LC×HC promoter levels (RPU 5/40/100) each duplicated in randomized order. Listed are the applied LC and HC promoter levels and the measured Titer (mg/L), VCD (cells/mL), and Viability (%) for mAb B.

Run	LC	HC	Titer (mg/L)	VCD (cells/mL)	Viability (%)
1	Medium (40)	Low (5)	2.09	2876666	79.4
2	Medium (40)	Medium (40)	10.3	2680000	78.3
3	High (100)	Low (5)	2.42	2426666	74.1
4	High (100)	Low (5)	2.42	2426666	74.1
5	Low (5)	Medium (40)	2.6	2220000	73.7
6	High (100)	High (100)	11.9	1766666	63.03
7	Low (5)	High (100)	2.57	2346666.6	72.23
8	Low (5)	Low (5)	2.48	1630000	70.5
9	Low (5)	Medium (40)	2.6	2220000	73.7
10	High (100)	Medium (40)	12.5	2600000	74.2
11	Medium (40)	Medium (40)	10.3	2680000	78.3
12	High (100)	High (100)	11.9	1766666	63.03
13	Medium (40)	High (100)	6.81	2476666.6	72.43
14	High (100)	Medium (40)	12.5	2600000	74.2
15	Medium (40)	Low (5)	2.09	2876666	79.4
16	Low (5)	Low (5)	2.48	1630000	70.5
17	Medium (40)	High (100)	6.81	2476666.6	72.43
18	Low (5)	High (100)	2.57	2346666.6	72.23

Supplementary Table S5. Design matrix and observed responses for mAb C (Custom D-optimal DoE). Eighteen runs cover the nine LC×HC promoter levels (RPU 5/40/100) each duplicated in randomized order. Listed are the applied LC and HC promoter levels and the measured Titer (mg/L), VCD (cells/mL), and Viability (%) for mAb C.

Run	LC	HC	Titer (mg/L)	VCD (cells/mL)	Viability (%)
1	Medium (40)	Low (5)	0	2720000	77.7
2	Medium (40)	Medium (40)	6.46	2240000	69.6
3	High (100)	Low (5)	1.13	1870000	64.5
4	High (100)	Low (5)	1.13	1870000	64.5
5	Low (5)	Medium (40)	1.57	1570000	70.1
6	High (100)	High (100)	0	1790000	57.3
7	Low (5)	High (100)	1.03	1950000	64.9
8	Low (5)	Low (5)	4.09	2380000	74.7
9	Low (5)	Medium (40)	1.57	1570000	70.1
10	High (100)	Medium (40)	4.53	1970000	64.4
11	Medium (40)	Medium (40)	6.46	2240000	69.6
12	High (100)	High (100)	0	1790000	57.3
13	Medium (40)	High (100)	5.23	2050000	71
14	High (100)	Medium (40)	4.53	1970000	64.4
15	Medium (40)	Low (5)	0	2720000	77.7
16	Low (5)	Low (5)	4.09	2380000	74.7
17	Medium (40)	High (100)	5.23	2050000	71
18	Low (5)	High (100)	1.03	1950000	64.9

Supplementary Table S6. Design matrix and observed responses for mAb E (Custom D-optimal DoE). Eighteen runs cover the nine LC×HC promoter levels (RPU 5/40/100) each duplicated in randomized order. Listed are the applied LC and HC promoter levels and the measured Titer (mg/L), VCD (cells/mL), and Viability (%) for mAb E.

Run	LC	HC	Titer (mg/L)	VCD (cells/mL)	Viability (%)
1	Medium (40)	Low (5)	2.71	2050000	64.4
2	Medium (40)	Medium (40)	10.31	2170000	67.7
3	High (100)	Low (5)	2.86	1940000	65.8
4	High (100)	Low (5)	2.86	1940000	65.8
5	Low (5)	Medium (40)	0	2390000	69.6
6	High (100)	High (100)	7.3	1770000	59.6
7	Low (5)	High (100)	0.48	1670000	61.4
8	Low (5)	Low (5)	2.46	2250000	68.5
9	Low (5)	Medium (40)	0	2390000	69.6
10	High (100)	Medium (40)	12.59	2250000	67.4
11	Medium (40)	Medium (40)	10.31	2170000	67.7
12	High (100)	High (100)	7.3	1770000	59.6
13	Medium (40)	High (100)	2.84	1830000	61.7
14	High (100)	Medium (40)	12.59	2250000	67.4
15	Medium (40)	Low (5)	2.71	2050000	64.4
16	Low (5)	Low (5)	2.46	2250000	68.5
17	Medium (40)	High (100)	2.84	1830000	61.7
18	Low (5)	High (100)	0.48	1670000	61.4

Supplementary Table S7. Model fit statistics for each antibody and response. Least-squares models (factors LC and HC treated as nominal) were fit to Titer (mg/L), VCD (cells/mL), and Viability (%) for mAbs B, C, and E. Reported are RMSE (typical prediction error, in response units), R^2 (variance explained), and the overall model p-value. All experiments were run with 18 observations (9 combinations × 2 replicates). p-values refer to the global model test.

	RMSE	R^2	P-value
**mAb B** Titer (mg/L)	2.34	0.78	0.0004
**mAb B** VCD (cells/mL)	3.18×10^5	0.54	0.029
**mAb B** Viability (%)	2.70	0.73	0.001
**mAb C** Titer (mg/L)	2.08	0.54	0.027
**mAb C** VCD (cells/mL)	1.43×10^5	0.81	0.0001
**mAb C** Viability (%)	1.98	0.92	0.0001
**mAb E** Titer (mg/L)	2.68	0.70	0.0022
**mAb E** VCD (cells/mL)	1.0×10^5	0.87	0.0001
**mAb E** Viability (%)	0.91	0.95	0.001

Supplementary Table S8. Effect tests (ANOVA) for HC and LC promoter strength across three antibodies. Least-squares models were fit for each response with factors treated as nominal. Shown are degrees of freedom (DF), sums of squares, F ratios, and p-values for LC and HC main effects. Responses are Titer (mg/L), VCD (cell/mL), and Viability (%).

		DF	Sum of Squares	F- Ratio	P-value
**mAb B** Titer (mg/L)	LC	2	124.2	11.32	0.0014
	HC	2	124.5	11.34	0.0014
**mAb B** VCD (cell/mL)	LC	2	1.17×10^{12}	6.15	0.013
	HC	2	2.81×10^{11}	1.47	0.264
**mAb B** Viability (%)	LC	2	126.03	8.62	0.0041
	HC	2	136.32	9.33	0.0031
**mAb C** Titer (mg/L)	LC	2	30.29	3.51	0.06
	HC	2	36.64	4.25	0.038
**mAb C** VCD (cell/mL)	LC	2	6.35×10^{11}	15.48	0.0004
	HC	2	4.84×10^{11}	11.79	0.0012
**mAb C** Viability(%)	LC	2	366.19	46.79	0.0001
	HC	2	182.80	23.36	0.0001
**mAb E** Titer (mg/L)	LC	2	134.85	9.39	0.003
	HC	2	84.14	5.86	0.0153
**mAb E** VCD (cell/mL)	LC	2	4.40×10^{10}	2.19	0.15
	HC	2	8.08×10^{11}	40.26	0.0001
**mAb E** Viability (%)	LC	2	17.42	10.47	0.002
	HC	2	172.44	103.7	0.0001
